# Appropriateness of Empirical Prescriptions of Ceftriaxone and Identification of Opportunities for Stewardship Interventions: A Single-Centre Cross-Sectional Study

**DOI:** 10.3390/antibiotics12020288

**Published:** 2023-02-01

**Authors:** Ana Gorgulho, Flávia Cunha, Elsa Alves Branco, Ana Azevedo, Francisco Almeida, Raquel Duro, Paulo Andrade, Nuno Rocha Pereira, Carlos Lima Alves

**Affiliations:** 1 Internal Medicine Department, Hospital de Cascais, Av. Brigadeiro Victor Novais Gonçalves, 2755-009 Cascais, Portugal; 2 Infectious Diseases Department, Centro Hospitalar Universitário de São João, 4200-319 Porto, Portugal; 3 Infectious Diseases Department, Hospital de Braga, 4710-243 Braga, Portugal; 4 Hospital Epidemiology Centre, Centro Hospitalar Universitário de São João, 4200-319 Porto, Portugal; 5 Department of Public Health and Forensic Sciences, Medical Education, Faculdade de Medicina da Universidade do Porto, 4200-319 Porto, Portugal; 6 EPIUnit, Instituto de Saúde Pública da Universidade do Porto, 4050-600 Porto, Portugal; 7 Laboratório para a Investigação Integrativa e Translacional em Saúde Populacional (ITR), Universidade do Porto, 4050-600 Porto, Portugal; 8 Infection and Antimicrobial Resistance Control and Prevention Unit, Hospital Epidemiology Centre, Centro Hospitalar Universitário de São João, 4200-319 Porto, Portugal; 9 Infectious Diseases Department, Centro Hospitalar de Tâmega e Sousa, 4564-007 Penafiel, Portugal; 10 Department of Medicine, Faculdade de Medicina da Universidade do Porto, 4200-319 Porto, Portugal

**Keywords:** ceftriaxone, antimicrobial stewardship, inappropriate prescribing

## Abstract

Third-generation cephalosporins are widely used due to the convenient spectrum of activity, safety, and posology. However, they are associated with the emergence of multidrug-resistant organisms, which makes them important targets for antimicrobial stewardship interventions. We aimed to assess the appropriateness of empirical prescriptions of ceftriaxone in a tertiary hospital. This cross-sectional study analysed empirical ceftriaxone prescriptions in January and June 2021. Patients under other antimicrobials 48 h before admission were excluded. The quality of ceftriaxone prescription was assessed regarding the initial appropriateness, duration of inappropriate ceftriaxone therapy, and missed opportunities for de-escalation. Of 465 prescriptions, 46.5% were inappropriate. The ceftriaxone prescription was inappropriate in 95.7% of lower respiratory tract infections (LRTI) globally and in nearly 40% of urinary tract infections (UTI) in medical and intensive care departments. Intensive care, internal medicine, and palliative care departments showed the highest number of inappropriate ceftriaxone prescriptions and longer length of inappropriate ceftriaxone prescriptions compared to the hospital’s average. Improvement of empirical ceftriaxone prescription in LRTI and urinary infections, adherence to local guidelines and de-escalation practices, and targeted interventions focusing on critical departments may significantly reduce the inappropriate empirical use of ceftriaxone.

## 1. Introduction

Third-generation cephalosporins (3GC) are widely used worldwide due to their broad-spectrum activity, favourable safety profile, and simple posology [1,2]. 

Especially for ceftriaxone, they are first-line antimicrobials in multiple clinical scenarios, including cholangitis, cholecystitis, pyelonephritis, urosepsis, acute meningitis, and gonococcal infections [3,4,5,6]. Notwithstanding, part of the large consumption of 3GC worldwide may represent situations in which their use could have been avoided, such as community-acquired pneumonia (CAP), acute cystitis, or surgical antimicrobial prophylaxis [4,7,8].

Their association with the emergence of multidrug-resistant organisms (MDRO) is concerning, namely with the colonisation and infection by extended-spectrum beta-lactamases (ESBL) being produced and fluoroquinolone-resistant Gram-negative bacteria, and also *Clostridioides difficile (C. diff)* infection [9,10,11,12,13,14]. Considering this, in 2021 WHO AWaRe classification, 3GC are considered a Watch group antibiotic [15].

Antimicrobial stewardship interventions directed at cephalosporin use have reduced inappropriate 3GC prescriptions and lowered the prevalence of ESBL-producing Gram-negative bacteria and *C. diff* infection [16,17,18].

Cephalosporins use has been increasing throughout European hospitals [1]. In particular, Portugal was the 11th largest consumer of cephalosporins in the EU/EEA in 2021, according to data from ECDC ESAC-Net [1]. As for resistance rates, in 2021, the prevalence of 3GC-resistant strains in Portugal was 45.0% for *Klebsiella pneumoniae* isolates and 13.1% for *Escherichia coli* [1,19].

Considering the broad use of ceftriaxone in the hospital sector and its negative impact on resistance rates, we aimed to assess the patterns and appropriateness of ceftriaxone empirical prescription and to identify opportunities for antimicrobial stewardship interventions in a tertiary care hospital in Portugal.

## 2. Results

During the study period, 465 prescriptions were included. Table 1 presents the patient’s demographic and clinical characteristics. Patients who received a prescription of ceftriaxone were mostly elderly, with 13.6% having been hospitalised in the previous three months and 15.1% having received antibiotic therapy in that period. More than 25% of the patients presented sepsis at the time of the prescription. Very few prescriptions were made in patients known to be colonised by MDRO (1.9%).

**Figure 1:**
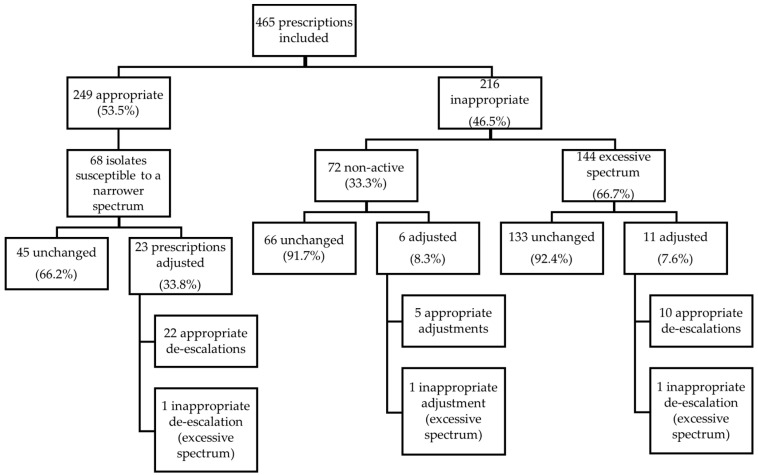
Appropriateness of initial prescriptions of ceftriaxone and de-escalation or further prescription changes.

The empirical prescription was inappropriate in 216 patients (46.5%): in 144 patients (66.7%) due to “excessive spectrum” and in 72 (33.3%) as “non-active” (Figure 1). Of the inappropriate prescriptions, only 17 (7.87%) were adjusted; most adjustments/de-escalations were appropriate (88.2%).

Strains with susceptibility to narrower spectrum antimicrobials were isolated in 68 patients among the 249 appropriate ceftriaxone prescriptions. De-escalation was performed in 23 cases (33.8%), with 22 de-escalations considered appropriate (Figure 1).

Ceftriaxone was prescribed for 2310 days in the study period, of which 1012 (43.8%) were inappropriate.

As shown in Table 2, the mean age of patients to whom empirical ceftriaxone was prescribed inappropriately was higher than those with an appropriate prescription. Inappropriate empirical ceftriaxone prescriptions were mostly made in the wards (*n* = 105), followed by the emergency department (*n* = 83). Most inappropriate ceftriaxone treatments were made in medical departments. The proportion of inappropriate prescriptions of ceftriaxone made in dwellers in long-term care facilities was higher than that of appropriate prescriptions of ceftriaxone.

Over two-thirds of inappropriate prescriptions of ceftriaxone were made for clinical syndromes covered by an internal guideline, almost as much as in the appropriate prescriptions of this drug. Situations not covered by internal guidelines were diverse and included skin and soft tissue infections, ear, nose and throat infections, ischemic colitis, and spontaneous bacterial peritonitis (and its prophylaxis). LRTI and UTI were the clinical situations with a local guideline with higher number of inappropriate use of ceftriaxone (Table 3). A higher proportion of inappropriate prescriptions was made on weekdays (49.5% of all weekdays prescriptions) than that of inappropriate prescriptions on weekends (38.8%; data not shown in the tables).

Most ceftriaxone prescriptions for treating lower respiratory tract infection (LRTI) were inappropriate across all departments (prescribed in 94 cases, 95.7% of which were inappropriate) (Table 4), as well as an important proportion of ceftriaxone prescriptions for urinary tract infection (UTI) in medical and intensive care departments (40.2% and 38.5%, respectively).

**Figure 2:**
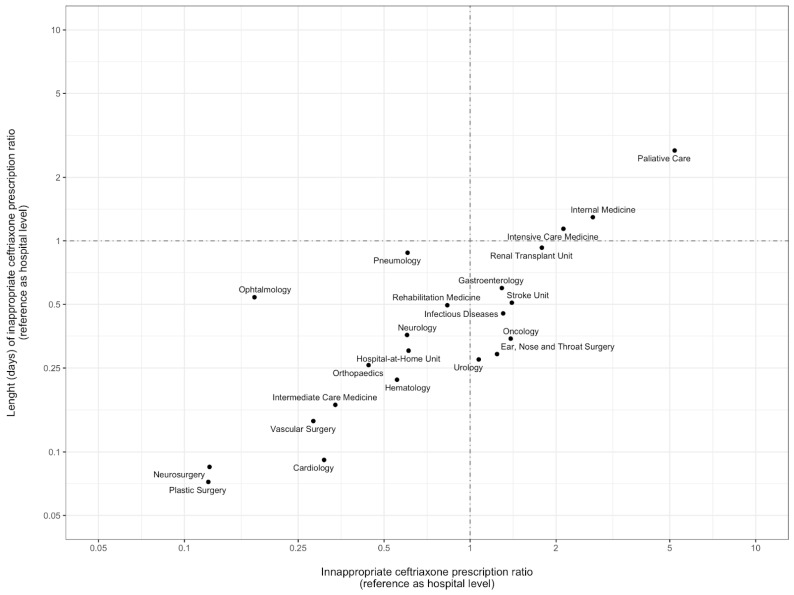
Evaluation of inappropriate empirical prescription of ceftriaxone, per department. Comparison between departments was performed using a ratio between the observed number of days of inappropriate ceftriaxone prescriptions and the expected number of days of inappropriate ceftriaxone prescriptions (with hospital level as reference). Dots represent the different hospital departments evaluated. The vertical and horizontal dot–dash lines represent the general hospital ratio (where the values observed correspond to the values expected for the number of inappropriate prescriptions and duration of inappropriate prescriptions). The axis values are represented in a logarithmic scale.

In Figure 2, the departments on the right side of the vertical line are those where the inappropriate empirical ceftriaxone prescription ratio is higher than the hospital average (with the hospital level as the reference). The intensive care, internal medicine, and palliative care departments showed both the highest ratios of inappropriate ceftriaxone prescription (112%, 169%, and 420% higher than expected with hospital level as reference, respectively) and length of inappropriate ceftriaxone prescription (with a duration of 14%, 29%, and 168% longer than anticipated with the hospital level as the reference, respectively).

## 3. Discussion

This study evaluated 465 patients with empirical ceftriaxone prescription over a two-month period. We studied ceftriaxone prescriptions from all hospital departments (ED, surgical, medical, and ICU) independently of their inherent variability, providing a more realistic picture of ceftriaxone prescription in routine clinical practice.

In a high proportion of patients (46.5%), this prescription was inappropriate, corresponding to 1012 days of inappropriate ceftriaxone use. These results are similar to those found in studies focused on the quality of antibiotic prescription, although there is high heterogeneity regarding prescription setting and methodology for quality assessments [20,21,22]. In approximately two-thirds of inappropriate prescriptions, ceftriaxone was considered to have an excessive spectrum of antimicrobial activity.

Lower respiratory infections were the most frequent syndrome with inappropriate ceftriaxone prescription across all major hospital departments. Ceftriaxone has not been associated with better outcomes in community-acquired pneumonia compared to amoxicillin–clavulanate and, since our local and national rate of resistance to aminopenicillins in the most common pathogens (*S. pneumoniae* and *H. influenzae*) is very low, our local guideline does not recommend ceftriaxone as first-line therapy in lower respiratory infections [23,24]

Besides lower respiratory infections, nearly 30% of prescriptions of ceftriaxone for the treatment of UTI in this study were inappropriate, despite local guidelines. From these, more than half (26/46) were cystitis. Even though this result is better than those found in other studies [25], it may be due to either unfamiliarity with the guideline or misdiagnosis. These might be topics for future evaluation and interventions.

The existence of institutional guidelines is not sufficient for changing behaviour, and this has been documented before [26,27]. An implementation strategy needs to be designed, ideally after identifying drivers of non-compliance.

Third-generation cephalosporins are not recommended for surgical prophylaxis [28] In our sample, these cases were mainly clean ear, nose, and throat surgeries and urologic procedures, with prolonged post-operative surgical prophylaxis. Extension of prophylactic antibiotics postoperatively in surgical wards is a significant concern, as no benefit has been demonstrated [14,29]. Even though this was a small portion of our inappropriate prescriptions, surgical prophylaxis indications and timings should be revisited and current practices should be re-evaluated.

Our study found that more than one-third (36.7%) of empirical ceftriaxone prescriptions in the ED were inappropriate. Considering its simplicity of use, either due to posology, absence of renal adjustment, or low level of toxicity, we would expect a relatively higher number of inappropriate prescriptions in this context. Interestingly, 92.1% of inappropriate prescriptions were not later modified, highlighting the importance of appropriate empirical prescriptions in the ED.

Palliative care, internal medicine and intensive care departments were found to have the highest ratios of inappropriate empirical prescription and the longer time to de-escalate or suspend inappropriate ceftriaxone prescriptions. The decision of antibiotic prescription in palliative care is often regarded as a form of symptom control, and there is a lack of clear guidelines in these circumstances. An extensive analysis of 3884 hospice patients has shown that 27% of patients received antibiotics in their last week of life, with only 15% of those having a documented infectious diagnosis [30].

Intensive care units are also challenging regarding antimicrobial stewardship due to the greater severity of illness, a lower threshold to initiate antibiotics, and frequent use of broad-spectrum antibiotics as empirical treatment for critically ill patients. These elevated ratios reflected non-adherence to current guidelines (local and international) and missed opportunities for de-escalation/suspension of inappropriate therapy. In a recent international study, antibiotic de-escalation within the three days after the empirical prescription was performed in only 16% of critically-ill patients, representing a low level of therapeutic tailoring; calculated rates at 5 and 7 days were slightly superior at 21% and 23%. In this study, the clinical cure was not negatively impacted in the de-escalation group [31].

Despite being under prospective audit and feedback stewardship interventions, renal transplant unit and urology results were surprising. About 83% of inappropriate prescriptions in the urology department were surgical prophylaxis, and 55% were prolonged post-procedure surgical prophylaxis. In addition, in both departments, empirical ceftriaxone was inappropriately prescribed in situations such as nosocomial pneumonia or surgical site infection.

Multiple interventions can be sought to improve these results. Education of healthcare staff, awareness of local antimicrobial resistance patterns, review of indications for antibiotic prescription and use/development of algorithms that support medical decisions may be options to improve antimicrobial stewardship. Specific audit and feedback interventions targeting ceftriaxone prescription in these departments can be prioritised, and department-specific reasons for inappropriate prescription should be clarified. A summary of the potential targets and antimicrobial stewardship interventions regarding ceftriaxone prescription is described in Table 5.

In our hospital, there has been an antimicrobial stewardship (AMS) and infection control program implemented since 2013 under the auspices of the National Program for the Prevention and Control of Infection and Antimicrobial Resistance. The AMS team carries out prospective audit and feedback interventions and participates in ward rounds in specific departments, mostly surgical departments (namely orthopaedics, vascular surgery, general surgery, cardiothoracic surgery, neurosurgery, urology, and plastic surgery) but also in the haematology and renal transplant units. Interestingly, most of the departments where the inappropriate empirical ceftriaxone prescription ratio was higher are outside the current scope of the AMS program. The AMS team also performs post-prescription validation of a group of antibiotics considered for restricted use across all hospital departments. This strategy does not cover 3GCs and could be an area of improvement.

The Global Point Prevalence Survey on Antimicrobial Consumption and Resistance (Global-PPS) has identified ceftriaxone as the most prescribed antibiotic for therapeutic use on adult wards worldwide, despite its classification as a “Watch” antibiotic by WHO AWaRe. It reported that ceftriaxone was still widely used as surgical prophylaxis in Eastern and Southern Europe and that 20% of ceftriaxone prescriptions were for pneumonia [32]. Global (or even national) studies on prescription quality, reasons for antibiotic misuse, and opportunities for targeted stewardship interventions are scarce.

Our study allowed us to have a global picture of ceftriaxone prescription across the hospital and identify problematic inappropriateness clusters. To the best of the authors’ knowledge, there are no previously published data from Portuguese health institutions regarding the qualitative evaluation of antimicrobial prescription. This analysis should be encouraged so that institutions can develop targeted and judicious stewardship strategies.

Several caveats need to be noted regarding the present study. Due to differences in prescription patterns in different settings, its single-centre design does not allow us to extrapolate our findings to design stewardship interventions elsewhere. Its cross-sectional design also further diminishes generalizability. Clinical information was retrieved from electronic medical records, with possible data quality issues due to the non-standardisation of medical notes. The adequacy of microbiological sampling to the clinical syndrome was not assessed, which may further compromise decisions. Our research focused on one single antibiotic. Therefore, the results do not represent the prescription pattern of each department/setting, either global antibiotic prescription, or other specific antibiotics or classes of antibiotics. The current research was not explicitly designed to evaluate patient outcomes. Further correlation with mortality, MDRO carriage and *C. diff* infection in patients with previous inappropriate ceftriaxone prescriptions, as well as the impact of antimicrobial stewardship interventions in these outcomes, should be pursued in future work.

In summary, we have found that almost half of empirical ceftriaxone prescriptions in our hospital were inappropriate during the study period. We postulate that improving empirical prescription in LRTI and urinary infections, adhesion to local guidelines, and de-escalation practices, microbiological sampling, and surgical prophylaxis use, and focusing on critical departments such as internal medicine, ICU, and palliative care could vastly decrease the inappropriate use of ceftriaxone in our setting.

## 4. Materials and Methods

### 4.1. Setting and Design

We performed a retrospective cross-sectional observational study at Centro Hospitalar Universitário de São João in Porto, Portugal. It is a tertiary care and referral centre in numerous areas, including bone marrow, kidney, and heart transplants, with a total capacity of 1105 beds and an active antimicrobial stewardship and infection control team.

### 4.2. Inclusion and Exclusion Criteria

We included all adult (≥18 years old) patients admitted to any hospital department for more than 24 h who were treated empirically with ceftriaxone for any indication in January and June of 2021. The decision to include one month of both summer and winter seasons aimed to increase representativeness by considering the variability in prescriptions motivated by infection seasonality. Patients under other antimicrobials 48 h before ceftriaxone administration or who started ceftriaxone as targeted therapy (after agent identification and susceptibility testing) were excluded.

### 4.3. Data Collection

A list of patients fulfilling the inclusion criteria was obtained from the hospital’s electronic information system. Electronic medical records were used to obtain data on diagnosis, clinical, and laboratory features, place and date of ceftriaxone’s initial prescription, allergies, prior hospitalisations, antibiotic prescriptions, known colonisation with MDRO, and date of ceftriaxone discontinuation. The number of daily admissions in each department was obtained from the hospital’s administrative database. The confidentiality of the data collected was maintained, and all patient identifiers were removed.

### 4.4. Outcomes

The primary outcome was the proportion of patients receiving inappropriate empirical ceftriaxone prescriptions. Secondary outcomes included the number of days of inappropriate ceftriaxone therapy during hospitalisation, the proportion of inappropriate empirical prescriptions for clinical conditions for which an internal hospital guideline was available and the appropriateness and proportion of de-escalation.

### 4.5. Definitions

#### 4.5.1. Empirical Ceftriaxone Prescription Appropriateness

A physician evaluated the appropriateness of each initial prescription. Compliance with internal guidelines, the patient’s history of allergies, prior antimicrobial therapy, and previous microbiology isolates were considered. Our hospital has local guidelines for pneumonia, urinary tract infections, intra-abdominal and biliary, central nervous system and osteoarticular infections, and surgical prophylaxis. In cases where the treatment indication did not fall under these guidelines, the appropriateness was independently evaluated by two infectious diseases/antimicrobial stewardship specialists and, in case of disagreement, by a third one. The most recent European recommendations for each infectious syndrome were used as a reference in these cases.

Inappropriate prescriptions were then defined as “non-active” or “excessive spectrum”: “non-active” if the antimicrobial did not have coverage for the common aetiology (example: ceftriaxone for empirical treatment of nosocomial pneumonia); “excessive spectrum” if the antimicrobial was not considered first-line but covered the common aetiology (example: ceftriaxone for CAP).

#### 4.5.2. Appropriate Suspension/De-Escalation of Ceftriaxone

We had access to the dates when susceptibility testing results were electronically available to clinicians for patients with positive cultures. Therefore, we could detect opportunities for de-escalation in patients for whom empirical ceftriaxone prescription was appropriate. When de-escalation was possible but not performed, the remaining days of the ceftriaxone prescription were considered inappropriate. The first 24 h after the issue of susceptibility results were not considered to account for the time between the availability of the results and the decision-making process by the physician. De-escalation from ceftriaxone, when it occurred, was also evaluated regarding appropriateness, following the above definitions of “non-active” and “excessive spectrum”.

#### 4.5.3. Days of Inappropriate Ceftriaxone

We calculated the length of inappropriate consumption of ceftriaxone by adding the number of days of ceftriaxone when the empirical prescription was inappropriate and the number of days that ceftriaxone was inappropriately maintained after susceptibility results were known (with 24 h margin).

### 4.6. Statistical Analysis

Descriptive statistics in frequency and percentages were used for variables such as demographic profile, disease conditions, and ceftriaxone prescribing patterns. Continuous data were reported as mean and standard deviation and compared using the Mann–Whitney test. Categorical data were reported as numbers and percentages and compared using the Chi2 test or Fisher test when necessary. To assess the possible targets for stewardship intervention regarding inappropriate ceftriaxone prescription, we compared the ratios of ceftriaxone inappropriate prescription and its duration per department, using the hospital’s global prescription ratios as a reference. Data on the number of ceftriaxone prescriptions, length of ceftriaxone prescription and number of admissions per department during the study period were collected. The probability of ceftriaxone prescription at the hospital level (total number of ceftriaxone prescriptions in the hospital/number of hospital admissions), probability of inappropriate ceftriaxone prescription at the hospital level (number of inappropriate ceftriaxone prescriptions in the hospital/total number of ceftriaxone prescriptions), and probability of inappropriate ceftriaxone prescription per hospital admission (probability of ceftriaxone prescription at the hospital level × probability of inappropriate ceftriaxone prescription at the hospital level) were calculated. To compare inappropriate ceftriaxone prescriptions between departments, we calculated, per department, a ratio between the observed number of inappropriate ceftriaxone prescriptions and the expected number of inappropriate ceftriaxone prescriptions (with hospital level as reference). Regarding the duration of the inappropriate prescription, we calculated a weighted mean of days of inappropriate ceftriaxone prescription in the hospital, using the number of inappropriate prescriptions. Using this weighted mean, we calculated the expected duration of inappropriate ceftriaxone prescription per department (weighted mean of days of inappropriate ceftriaxone prescription*number of admissions with ceftriaxone prescription). A comparison between departments was performed using a ratio between the observed number of days of inappropriate ceftriaxone prescriptions and the expected number of days of inappropriate ceftriaxone prescriptions (with hospital level as reference). The scatterplot in Figure 2 represents the ratios described. Data were analysed using RStudio version 2022.07.1, RStudio, PBC, Boston, MA, USA.

## Figures and Tables

**Table 1:** Patient’s demographic and clinical characteristics.

	All Prescriptions *n* = 465
Male sex—*n* (%)	249 (53.5)
Age, years—mean (SD)	69.7 (17.7)
Long-term care facility residency—*n* (%)	28 (6.0)
Transfer from another hospital—*n* (%)	43 (9.2)
Known antibiotic allergy—*n* (%)	18 (3.9)
Hospitalisation in the previous 3 months—*n* (%)	63 (13.6)
Antibiotic therapy in the previous 3 months—*n* (%)	70 (15.1)
Presence of sepsis—*n* (%)	124 (26.7)
Intensive care admission—*n* (%)	58 (12.5)
Isolated pathogens in the previous 3 months—*n* (%)	29 (6.2)
Colonisation by MDRO—*n* (%)	9 (1.9)

**Table 2:** Characteristics of empirical ceftriaxone prescriptions, according to appropriateness.

	Appropriate Prescription *n* = 249	Inappropriate Prescription *n* = 216	*p* Value
Sex—*n* (%) Male			
	130 (52.2)	119 (55.1)	0.597
Age (mean ± SD), y	66.5 (18.1)	73.4 (16.5)	<0.001
Long-term care facility residency—*n* (%)	10 (4.0)	18 (8.3)	0.079
Transfer from another hospital—*n* (%)	25 (10.0)	18 (8.3)	0.636
Hospitalisation department during the treatment—*n* (%)			
Medical	85 (34.1)	131 (60.6)	Ref.
Surgical	146 (58.6)	58 (26.9)	<0.001
Intensive Care	18 (7.3)	27 (12.5)	0.931
Known antibiotic allergy—*n* (%)	14 (5.6)	4 (1.8)	0.063
Intensive care admission—*n* (%)	28 (11.2)	30 (13.9)	0.394
Presence of sepsis—*n* (%)	77 (30.9)	47 (21.8)	0.034
Previous antibiotic therapy—*n* (%)	36 (14.5)	34 (15.7)	0.798
Hospitalisation in the last 3 months—*n* (%)	43 (17.3)	20 (9.3)	0.017
Colonisation by MDRO—*n* (%)	3 (1.2)	6 (2.8)	0.314
Defined hospital guideline—*n* (%)	180 (72.3)	150 (69.4)	0.424
Previously isolated pathogens—*n* (%)	13 (5.2)	16 (7.4)	0.435
Microbiological sampling—*n* (%)	195 (78.3)	160 (74.1)	0.335
Day of antibiotic initiation—*n* (%)			
Weekday	167 (67.1)	164 (75.9)	Ref.
Weekend	82 (32.9)	52 (24.1)	0.036
Setting of empirical antibiotic prescription—*n* (%)			
ED	143 (57.4)	83 (38.4)	Ref.
Ward	96 (38.6)	105 (48.6)	0.001
ICU	8 (3.2)	19 (8.8)	0.001
OR	2 (0.8)	9 (4.2)	0.004
Aim of antibiotic prescription—*n* (%)			
Treatment	249 (100)	181 (83.8)	Ref.
Prophylaxis	0 (0.0)	35 (16.2)	<0.001
Ceftriaxone duration (mean ± SD), days	5.1 (3.6)	4.4 (4.2)	0.002

ED—emergency department; ICU—intensive care unit; MDRO—multidrug-resistant organisms; OR—operating room; SD—standard deviation.

**Table 3:** Appropriateness of empirical ceftriaxone prescriptions for clinical conditions with a defined hospital protocol.

Hospital Protocol	Appropriate Prescription N = 180	Inappropriate Prescription N= 150
Intra-abdominal and biliary infections—*n* (%)	48 (100)	0 (0)
LRTI—*n* (%)	4 (4.3)	90 (95.7)
UTI—*n* (%)	119 (72.1)	46 (27.9)
Osteoarticular infection—*n* (%)	6 (85.7)	1 (14.3)
CNS infection—*n* (%)	3 (100)	0 (0)
Surgical prophylaxis—*n* (%)	0 (0)	13 (100)

CNS—central nervous system; LRTI—lower respiratory tract infection; UTI—urinary tract infection.

**Table 4:** Appropriateness of empirical ceftriaxone prescriptions per infection syndrome and department.

Hospitalisation Department	Infectious Syndrome	Appropriate Prescription *n* (%)	Inappropriate Prescription *n* (%)
Medical (*n* = 215)	Abdominal	10 (100)	0 (0)
	Biliary	1 (100)	0 (0)
	Lower respiratory	3 (4.3)	66 (95.7)
	Urinary	55 (59.8)	37 (40.2)
	Central nervous system	4 (100)	0 (0)
	Osteoarticular	1 (100)	0 (0)
	Skin and soft tissue	5 (83.3)	1 (16.7)
	Other	6 (18.8)	26 (81.2)
Surgical (*n* = 170)	Abdominal	29 (96.7)	1 (3.3)
	Biliary	28 (100)	0 (0)
	Lower respiratory	1 (14.3)	6 (85.7)
	Urinary	60 (92.3)	5 (7.7)
	Central nervous system	1 (100)	0 (0)
	Osteoarticular	6 (100)	0 (0)
	Skin and soft tissue	10 (100)	0 (0)
	Other	11 (47.8)	12 (52.2)
Intensive Care (*n* = 45)	Abdominal	3 (75)	1 (25)
	Biliary	1 (100)	0 (0)
	Lower respiratory	0 (0)	18 (100)
	Urinary	8 (61.5)	5 (38.5)
	Central nervous system	5 (100)	0 (0)
	Osteoarticular	0 (0)	1 (100)
	Skin and soft tissue	1 (100)	0 (0)
	Other	0 (0)	2 (100)

**Table 5:** Potential opportunities for antimicrobial stewardship interventions regarding ceftriaxone prescription.

Problem Identified	Potential Stewardship Intervention(s)
Inappropriate empirical prescription in lower respiratory and urinary tract infections	Understanding the drivers for excessive spectrum prescription in these syndromes Intensification of feedback regarding local patterns of antimicrobial resistance of the primary pathogens Post-prescription review of ceftriaxone use in the hospital
Non-adherence to internal guidelines	Raising awareness for the existence of local guidelines Promoting adherence to local guidelines regarding empirical antibiotic therapy
High ratios of inappropriate prescription in palliative care, internal medicine and intensive care departments, regarding both empirical prescription and duration of inappropriate therapy	Identifying drivers for inappropriate empirical prescription in these specific departments Audit and feedback interventions Targeted interventions at the level of these prescribers, such as antimicrobial stewardship ward rounds
Ceftriaxone as surgical prophylaxis	Identification of surgical procedures with inappropriate prophylaxis Promoting adherence to local surgical prophylaxis guidelines
Missed opportunities for de-escalation	Implementation of electronic alerts for possible narrowing of the antimicrobial spectrum Training of prescribers regarding the importance of frequent re-evaluation of microbiology results and antimicrobial prescription Audit and feedback interventions
Lack of microbiological sampling	Assessing potential difficulties to microbiological specimen collection (logistical/infrastructural, lack of training, among others) Training of healthcare workers

## Data Availability

Not applicable.
